# The clinical range and management of spontaneous rupture of the pathological malarial spleen (SRPMS): A short case series from Sudan

**DOI:** 10.1016/j.ijscr.2025.110818

**Published:** 2025-01-06

**Authors:** Eltahir Ahmed Eltahir, Mohamed Ahmed M. Ibnouf, Mohammed M.A.M. Ibnouf, Mohamed H. Ahmed, Mohammed Haroon Imam, Atif Ahmed

**Affiliations:** a Surgery University of Elfashir, Darfur, Sudan; b Faculty of Medicine and Health Sciences, Omdurman Islamic University, Sudan; c State Ministry of Health, North Darfur State, Sudan

**Keywords:** Rupture spleen, Malaria, Atraumatic spleen, Pneumococcal vaccine

## Abstract

**Introduction:**

Spontaneous rupture of the pathological malarial spleen (SRPMS) is a rare condition with a mortality rate among travelers of approximately 38 %, whereas it was around 10 % for local citizens. The mortality rate for overwhelming post-splenectomy sepsis was reported to be about 50 %.

**Methods:**

A retrospective study was conducted from febraury2022 to July 2022. Baseline clinical presentations, management, and outcomes were recorded for analysis.

**Results:**

We present a brief series of eight patients with (SRPMS). Of them, five had successful conservative treatment, while three underwent splenectomy. Only one of them received Pneumococcal vaccine.

**Conclusion:**

SRPMS is a rare complication of malaria infection, it can be managed non-surgically, and splenectomy is necessary for unstable patients. Post-surgery vaccination remains crucial to prevent severe infections.

## Highlights

• Spontaneous rupture of the spleen caused by malaria (SRPMS) is a rare and serious condition.
• Quick and personalized care can make a big difference in saving lives.
• Many patients can recover with non-surgical treatments.
• Vaccines like Pneumovax are crucial after spleen removal to protect against severe infections.
• Ongoing care and prevention strategies are essential for a healthy recovery after surgery.

## 1 Background

Spontaneous rupture of the pathological malarial spleen (SRMS) is uncommon, yet it is a serious condition, mainly affecting endemic areas and travelers, occurring in an estimated 2 % of cases [1,2]. The precise incidence of SPRMS is difficult to determine primarily because of underreporting; however, it carries high mortality rates, particularly among travelers who have limited immunity [3]. Malaria-induced splenomegaly compromises the splenic capsule, making it susceptible to rupture even in the absence of obvious trauma [4]. Clinical manifestations can vary, displaying symptoms such as abdominal pain, fever, and hemodynamic instability, which require immediate diagnosis and intervention [5]. Imaging techniques, particularly ultrasound, are critical in diagnosing splenic rupture, and treatment approaches range from conservative care to emergency splenectomy based on the patient's condition [6]. Our case series was designed following the ‘SCARE’ guideline for case series [7].

## 2 Case presentations

Four of the eight diagnosed cases of SRPMS were males. The median age was 51, with a mean (±SD) age of 44 (±15.66) years and a range (23–61) years. The predominant symptoms were fever and pain. Details of the clinical presentation are summarised in Table 1.

**Table 1:** The symptoms and signs of our patients during presentation.

Fever	7 patients
Pain: Abdominal pain with left shoulder pain	3 patients
Left lower chest pain	5 patients
Vomiting	3 patients
Duration of symptoms: <1 week	1 patient
1–2 weeks	3 patients
>2 weeks	4 patients
Pulse on presentation: >100 beats/min	2 patients
90–100 beats/min	4 patients
<90 beats/min	2 patients
BP: Systolic > 100 mm Hg	3 patients
Systolic < 100 mm Hg	5 patients
Pallor:	5 patients
Temperature: 37.3–38 °C	4 patients
38.1–39.9 °C	3 patients
>39.9 °C	1 patient

All eight patients tested positive for malaria in their peripheral blood film, which displayed Trophozoites (ring stage) of Plasmodium falciparum. Upon presentation, the mean (±SD) haemoglobin level was 7.4 (±2.02), with a median of 8 and the range (4.5–10) gm/dl. The mean (±SD) white blood cell count was 6.587 × 10^9^/L (±2.41) with a range of 5000 × 10^9^ to 24.000 × 10^9^/L. Five patients had thrombocytopenia, with a mean (±SD) platelet count of 70.212 × 10^9^/ml (±63.132 × 10^9^/L). The ultrasound report of the abdomen indicated splenic infarction and peri-splenic hematoma in four cases and peri-splenic hematoma with hemoperitoneum in the remaining cases. The size of the spleen ranged from 14 to 17 cm; however, the status of the splenic vessels was not reported.

**Fig. 1:**
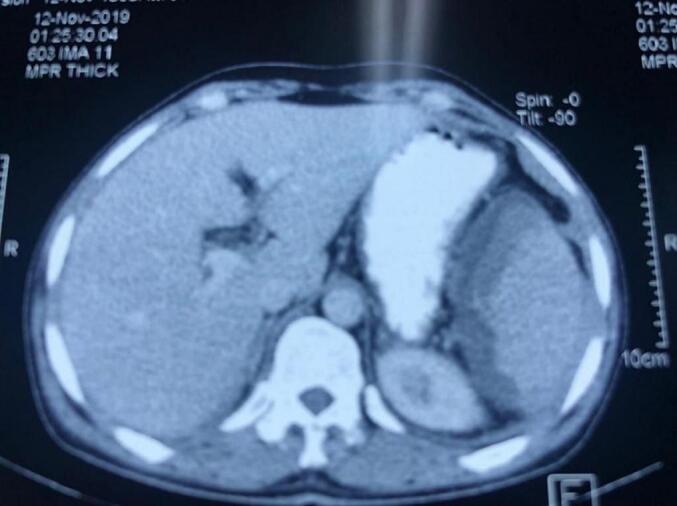
The CT scan reveals a peri splenic hematoma and hemoperitoneum.

Four quadrant taps revealed frank blood in four patients, two of them were hemodynamically unstable and their consciousness was deteriorating. One patient underwent a CT scan that demonstrated peri-splenic hematoma and hemoperitoneum, as illustrated in Fig. 1.

**Fig. 2:**
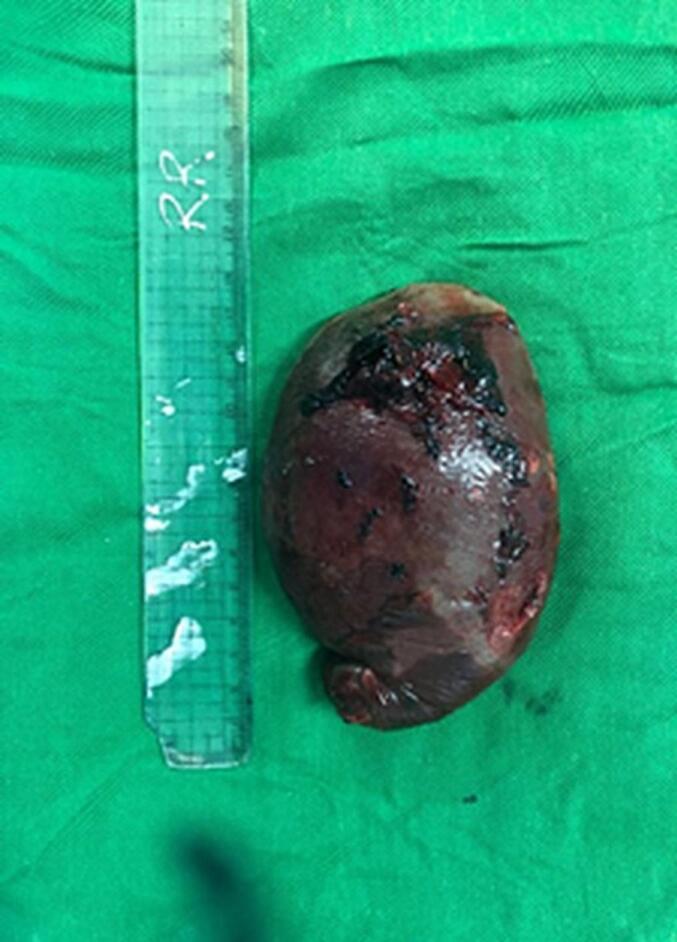
Shows laceration in the capsule of the spleen.

All patients were resuscitated in the Emergency Room with normal saline and received artemether 80 mg as intramuscular injections. The patients were transferred to a high-dependency unit, where they received blood transfusions that ranged from two to six units. Five patients showed progressive recovery. Three patients continued to experience low blood pressure. These patients underwent emergency laparotomy and splenectomy. Intraoperative findings indicated the presence of frank hemoperitoneum with clotted blood surrounding their lacerated enlarged spleen (Fig. 2).

Only one patient received an anti-pneumococcal vaccine on the second day of surgery. The three patients who had laparotomy and splenectomy recovered and were discharged with a prescription for oral Amoxycillin, to be taken at a dose of one gram daily for one month. Follow-up was for one week for one patient, two weeks for five patients, and eight weeks for two patients.

Histopathological analysis of the spleen revealed a laceration in the splenic capsule and malaria pigment with phagocytes in the swollen white pulp.

## 3 Patients and methods

The study included eight patients diagnosed with spontaneous rupture of pathological malarial spleen from February 2022 to July 2022. The data collected were age, sex, symptoms (fever, left upper abdominal pain), vital signs (hypotension, tachycardia, and respiratory distress), laboratory findings (anaemia, thrombocytopenia, and blood film microscopic and/or serology examination for plasmodia), ultrasound and/or CT scan report on splenic infarction and/or rupture, the modality of treatment and its outcomes, chemoprophylaxis, and or post-splenectomy vaccination.

## 4 Statistical analysis

Relevant data were fed to the Statistical Package for Social Sciences (SPSS) version 26. Because of the small number of cases, descriptive statistical analysis was done. Means and standard deviations were computed where appropriate. The work has been reported as being in line with the PROCESS criteria.

## 5 Discussion

The first case of a spontaneous splenic rupture was reported in 1874 by Edward Atkinson [8], and in 1958 the criteria for diagnosing PSRMS was described [9]. One of these criteria is that there must be no identifiable cause for the splenic rupture, such as infection, malignancy, medications, or medical interventions [10]. Malaria is the leading tropical infection that causes spontaneous splenic rupture [11]. Plasmodium vivax is the commonest reported plasmodium that causes SPRMS, however, Plasmodium falciparum was the causative organism in our eight cases the reason being the high endemicity of P. falciparum malaria in Sudan [12,13]. In the report of Imbert P. et al., of 55 cases of SPRMS, plasmodium falciparum was found in 26 patients, while *P. vivax* was diagnosed in 23 patients [1].

Five out of our eight patients had severe malaria according to the description of severe malaria reported in the WHO guidelines [14]. However, virology tests to rule out other causes of splenic rupture and histopathological examinations are not available in the remote region of Darfur State. Diagnosing splenic rupture in patients with malaria is complex due to overlapping symptoms with the underlying illness, which can lead to diagnostic delays and increased mortality rates. Without a preceding history of trauma, splenic rupture may not be suspected, however, minor abdominal trauma, straining or cough might trigger the rupture of a pathologically enlarged spleen [15]. The key indicator to diagnose SPRMS is the sudden abdominal pain in patients associated with low blood pressure. However, tachycardia and anaemia remain alarming signs [16]. Nevertheless, abdominal guarding and tenderness may not always be present, and the spleen might not be palpable. The signs rarely encountered include Kehr's sign (patient feels left shoulder pain in the presence of left subdiaphragmatic blood) [17], and Balance's sign (a mass or fixed dullness in the left upper quadrant) [17]. Physical examination often reveals signs of abdominal tenderness, absent bowel sounds, and hypovolemia, which can progress to shock if not treated promptly. Management relies on the patient's hemodynamic status. Conservative management is considered the gold standard of treatment for hemodynamically stable patients, while, operative management is currently the standard practice for hemodynamically unstable patients. Matthew Lukies and Warren Clements in their systematic review which was published in Feb 2024, reported that 30 patients were treated with splenic artery embolisation, 163 with splenectomy and only three with splenorrhaphy with no mortality [18]. However, our current facilities like other tropical African countries are poor and lack the appropriate facilities, for laparoscopy and/or splenic artery embolisation [19].

To prevent the overwhelming post-splenic infection (OPSI), conservative watchful management to preserve the function of the spleen is justifiable; however, in the hemodynamically unstable patient emergency splenectomy remains the treatment of choice to be followed by Pneumococcal, H. influenza, and meningococcal vaccination [20]. Unfortunately, these vaccines may not be available in low-resource countries. This fact explains that only one out of our eight cases received the pneumococcal vaccine.

## 6 Conclusion

Spontaneous rupture of pathological malarial spleen SRMS is a rare and serious complication, The outcomes of our patients' management highlight the necessity for early diagnosis and treatment with an inclination towards conserving the splenic function for the patient's future well-being as a cornerstone clinical governance.

## 7 Limitations of this study

1- The limited number of patients in this report dictated the implementation of descriptive statistics.
2- The poor resources hindered vaccination to prevent the overwhelming Post splenectomy infection except for one patient.
3- Because the patients' residences were in remote areas in the region of Dar For, where access to communication was limited. Additionally, these regions of western Sudan have been affected by armed conflict since 2003, further complicating the ability to maintain regular follow-up.

## Ethical clearance

Not applicable.

## CRediT authorship contribution statement

Conception and design of the study: MohamedAhmed M Ibnouf, Eltahir A Eltahir.

Acquisition of data: Eltahir A. Eltahir, Mohammed MAM Ibnouf, Mohamed H Ahmed, and Mohammed Haroon.

Analysis and interpretation of data and literature review: Eltahir Ahmed Eltahir, and Atif Ahmed.

Drafting the article: MohamedAhmed M Ibnouf, Eltahir A. Eltahir.

## Ethical approval

Ethical approval was obtained from the local ethical committee/research

Research Ethics Committee, Faculty of Medicine, Omdurman Islamic University

(Registration number 125)

## Guarantor

Eltahir Ahmed Eltahir.

## Research Registration Number

Not applicable.

## Author agreement statement

1) We the undersigned declare that this manuscript is original, has not been published before, and is not currently being considered for publication elsewhere.
2) We confirm that we have read and approved the manuscript and are the only persons responsible for its authorship.
3) We understand that the Corresponding Author is the sole contact for the Editorial process. He is responsible for communicating with the other authors about progress and final approval of proofs.
4) We transfer the copy to the international Journal of surgery Case Reports.

## Provenance and peer review

Not commissioned, externally peer-reviewed.

## Consent for publication

Written informed consent for the publication of this case report and its accompanying images was taken. A copy of the written consent was available for review by the Editor-in-Chief of this journal on request.

## Funding

No funding was received for this study.

## Declaration of competing interest

The authors declare no conflict of interest.
